# Gorham-Stout Disease: A Case Report and Review of the Literature

**DOI:** 10.7759/cureus.38756

**Published:** 2023-05-09

**Authors:** Bedirhan Albayrak, Şafak Aydın Şimşek, Tolgahan Cengiz, Oğuzhan Muslu, İsmail Büyükceran, Hüseyin Sina Coşkun, Nevzat Dabak

**Affiliations:** 1 Department of Orthopaedics and Traumatology, Ondokuz Mayıs University, Faculty of Medicine, Samsun, TUR; 2 Department of Orthopaedics and Traumatology, Hatay Training and Research Hospital, Hatay, TUR

**Keywords:** gorham-stout disease, cystic angiomatosis of bone, idiopathic multicentric osteolysis, disappearing bone disease, massive osteolysis, vascular anomaly

## Abstract

Gorham-Stout disease causes gradual bone loss (osteolysis) due to an abnormal overgrowth of lymphatic vessels. This rare disease is usually seen in younger people. The etiopathology of Gorham-Stout disease remains unclear. The disease is pathologically characterized by the proliferation of the vascular or lymphatic vessels and, finally, bone matrix destruction. These pathological changes lead to the presence of massive osteolysis on plain radiographs. Thus, plain radiograph findings may lead physicians to consider tumoral conditions, especially metastasis. There are several other conditions on the differential diagnosis list of massive osteolysis, such as metabolic, infectious, malignant, and immunological conditions. After excluding all possible disorders, the disease can be considered in the differential diagnosis. The treatment of the disease is symptom-based, but there is no consensus. Pharmacological methods should be considered first-line treatment. If there is no regression in the course of the disease despite pharmacological treatment, radiotherapy and resection arthroplasty are the treatment of choice in the later stages. In this case report, we present a case of Gorham-Stout disease, which was treated by pharmacological methods. During the one and half year follow-up, the local control of the disease was achieved without any surgical intervention.

## Introduction

Gorham-Stout disease (GSD), known as disappearing bone disease or massive osteolysis, was first reported by Jackson in 1838 [1]. Gorham and Stout described the clinical and pathological features of the disease in a series of 24 patients in 1955 [2]. Although almost 350 cases have been reported in the literature, its pathophysiology and etiology remain unknown. The disease usually occurs at younger ages. The symptoms of the disease are swelling in the joints, pain, and progressive deformity. For the diagnosis of GSD, metabolic, endocrine, infectious, malignant, and immunological conditions that may lead to bone resorption should be excluded [3]. Minor trauma history was also recorded in some of the cases [4].

Regarding the pathological examination, with the proliferation of vascular and lymphatic vessels, the bone matrix is destroyed, the fibrous tissue replaces the bone marrow, and the trabeculae become thinner. It can also be confused with lymphangiomatosis.

Although GSD can develop in any part of the skeletal system, it is usually located in the shoulder, pelvic girdle, and skull [5]. The pelvic girdle is challenging for the surgeon when surgical intervention is considered due to its complex anatomy. When it occurs in the extremities, it can cause a painful range of motion and weight-bearing pain.

In this case report, we aim to present a rare case of GSD located around the knee and review the relevant literature.

## Case presentation

A 66-year-old female patient was admitted to the physical therapy and rehabilitation department for two years due to pain in the right knee. She was referred to the orthopedics outpatient clinic after lytic areas were observed in the right knee on plain radiographs.

The patient’s physical examination demonstrated that the affected knee’s range of motion was slightly painful, and the flexion range was 0-90°. Edema, swelling, erythema, and warmth were absent in the affected knee, but slight valgus deformity was detected. She could mobilize unassisted. The neurovascular examination was regular. On blood tests, rheumatological parameters, such as rheumatoid arthritis, anti-cyclic citrullinated peptide, anti-nuclear antibodies, anti-neutrophil cytoplasmic antibodies, and whole blood, biochemistry, coagulation, calcium, alkaline phosphatase, parathyroid hormone, and vitamin D values were found to be in the normal range.

**Figure 1:**
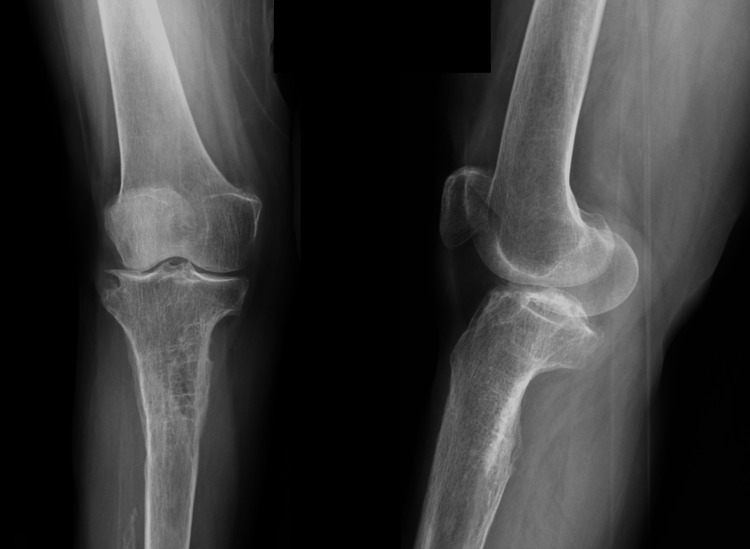
Plain radiographs showing lytic bone lesions in the proximal tibia (partially) and fibula. Note that the fibula is almost invisible on the plain radiographs.

**Figure 2:**
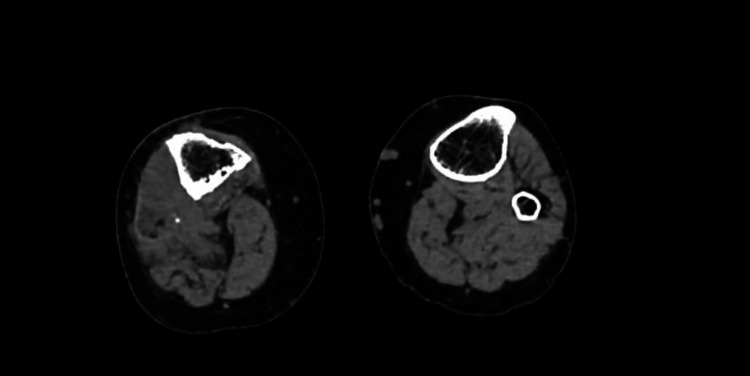
Irregularity and resorption areas are observed in the entire fibula and on both cortices of the tibia when compared to the left side.

**Figure 3:**
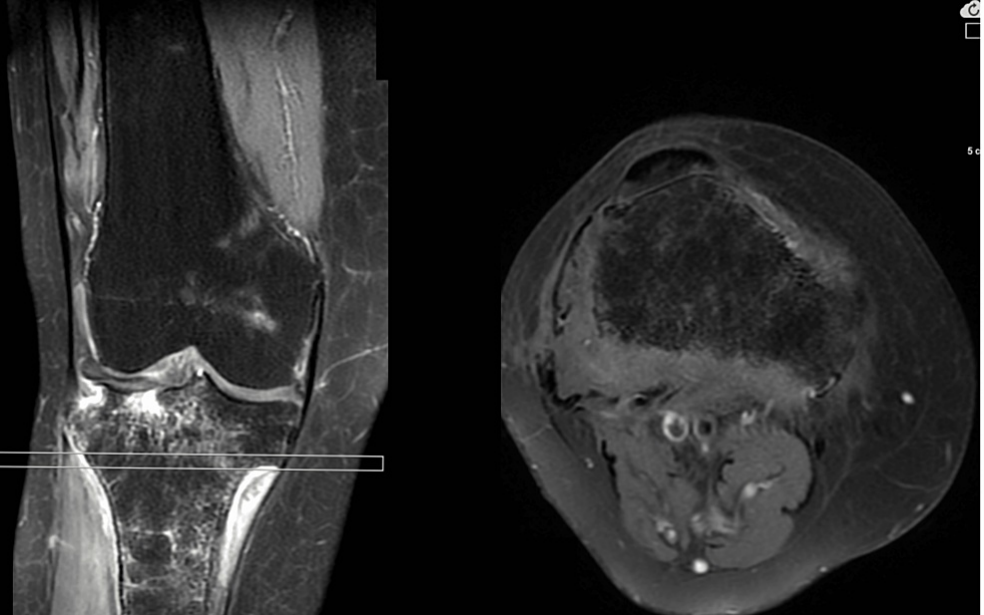
On the coronal and axial T2-weighted MRI images of the patient, hypointense osteolysis in the proximal fibula and medial and lateral resorptive changes with bone marrow edema in the tibia are observed.

**Figure 4:**
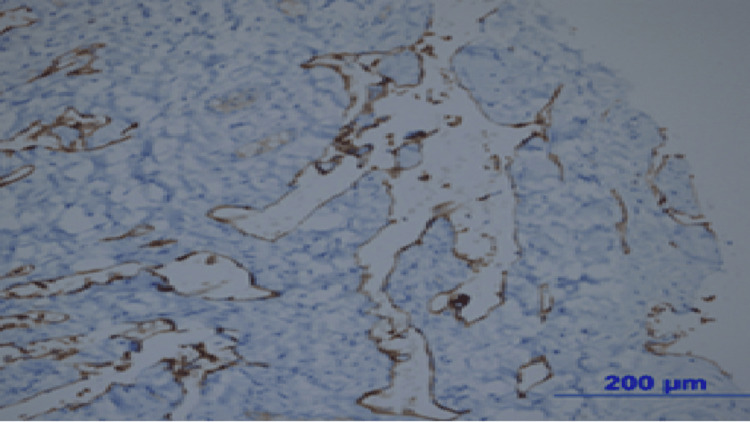
Numerous enlarged capillary structures are observed in the connective tissue (hematoxylin and eosin, ×200).

Upon detection of lytic bone lesions on plain radiographs (Figure 1), computed tomography (CT) and magnetic resonance imaging (MRI) were performed for detailed examinations (Figures 2, 3). A positron emission tomography (PET) was ordered to determine if there was a primary tumor or metastasis site. No pathological findings were found in the brain and thorax CT scans and PET images. As a result of these examinations, a biopsy was planned for the patient after a multidisciplinary discussion. On pathological examination, several dilated capillary vascular structures were detected in the connective tissue (Figure 4). After these clinical, radiological, and pathological assessments, other possible differential diagnoses were excluded, and the patient was diagnosed with GSD. The patient was informed about the treatment options. Finally, pharmaceutical treatment methods were chosen as the initial treatment. The patient was treated with non-steroidal anti-inflammatory drugs, supplementation of vitamin D, and bisphosphonate. During the third-month follow-up, the patient’s symptoms were relieved. The patient was clinically followed up for one and a half years and no progress of the disease was noted.

## Discussion

GSD is a diagnosis of exclusion confirmed after clinical and radiological examination and histopathological studies of the affected region. This bone disease is idiopathic, spontaneous, rare, and accompanied by massive bone osteolysis without new bone formation. GSD has no difference in terms of age, race, and gender. The elderly may have this disease, but it is usually seen in younger people [1]. Minor trauma may cause the disease, but etiological factors are controversial [2].

The prognosis of the disease depends on neurological and pulmonary complications. A total of 350 cases have been reported in the literature. Although any bone in the skeletal system can be affected by this disease, the maxillofacial area is the most affected region. The role of osteoclasts, angiogenesis/lymphangiogenesis, and disruption of osteoblast functions are the three main etiopathological features [2,3]. It has been suggested that bone destruction is due to local vascular angiomatosis. This increase in vascularization disrupts the osteoblast-osteoclast balance and causes an increase in bone resorption. Devlin et al. suggested that increased osteoclast activity associated with increased serum interleukin-6 contributes to bone resorption in GSD [2,6].

Localized pain, weakness in involved extremities, swelling, and skeletal deformities are dominant symptoms [4,5]. Neurological symptoms include paraplegia, rhinorrhea, and hearing disorders. Respiratory symptoms include chylothorax, dyspnea, and pleural effusion, and skin problems include lymphangiomatous and malformations. Laboratory findings are not specific, but alkaline phosphatase and IL-6 levels can increase [7]. In this case, our patient had localized knee pain, and laboratory findings showed no abnormality.

The partial and total absence of junctional bone and absence of sclerosing or osteoblastic reactions are diagnostic radiographic features of bone disappearing disease. On plain radiographs, subcortical and intramedullary radiolucent foci are seen in the initial stage, and when the clinical picture settles, characteristic osteolysis (without osteosclerosis or periosteal reaction) is noted. Bone loss and resorption are observed on MRI and may help demonstrate soft tissue involvement. The lesion appears hypointense on T1-weighted MRI and hyperintense on T2-weighted MRI [8]. MRI can clearly show increased contrast and vascular and/or lymphatic vessels within the bone in the region of active osteolysis. Bone scintigraphy shows increased uptake in areas of increased lymphatic and vascular proliferation and decreased uptake in the osteolytic region of lost bone [6]. In this case, osteolysis in the proximal fibula and resorptive appearances in the proximal tibia were detected on both CT and MRI.

Heffez et al. described eight clinical and histopathological criteria for diagnosing GSD. Positive biopsy for angiomatosis, absence of tumor and cellular atypia, absence of osteoblastic response, signs of local progressive resorption, non-expansive non-ulcerative lesion, absence of visceral involvement, osteolytic radiographic pattern, and absence of underlying genetic, immunological, endocrinological, metabolic, oncological, rheumatological etiologies [9]. According to this definition of the disease, all criteria were met by our patient.

Diagnosing GSD can be challenging and requires the exclusion of all other potential causes of osteolysis, such as cancer, infection, and hereditary diseases. Major disorders reported in the differential diagnosis are lymphangiomatosis, multiple myeloma, metastatic lytic lesions from an unknown primary tumor, Hajdu-Cheney syndrome, Paget’s disease, rheumatoid arthritis, fibrous dysplasia, Langerhans cell histiocytosis, Winchester syndrome (Hardegger type V classification), carpal-tarsal osteolysis, and eosinophilic granulomatosis. There was no primary site of the tumor in our patient and no elevation of C-reactive protein and sedimentation to suggest infection. No erythema and warmth in the extremities were observed on the physical examination. Normal rheumatologic laboratory findings and pain patterns excluded the possibility of rheumatologic disease. Pathologic findings favoring enlarged capillary vessels excluded other pathologies, such as multiple myeloma and lymphangiomatosis.

There is no consensus on the treatment of GSD. Pharmaceutical and surgical treatment and radiotherapy have been tried to treat this disease. It has shown a solid anti-angiogenic effect with the synergistic use of bisphosphonates and alpha-2b interferons in pharmacological treatment [10,11]. Bisphosphonates, calcium, and vitamin D have been used since the introduction of the first case. Octreotide, bevacizumab, taxol, vincristine, sunitinib, calcitonin, and propranolol have also been tried in patients who do not respond to conventional first-line therapy. Sirolimus has recently been used and satisfactory results have been obtained. Vascular anomalies have variable clinical features, and anti-angiogenic agents used in these diseases are included in the treatment for GSD. Sirolimus appears to have a promising stabilization effect on malignant vascular tumors, but it is unclear. In patients with simple lymphatic malformation, the mammalian target of rapamycin inhibition is also an alternative and has been shown to have a role in GSD [12]. Radiotherapy can be used preoperatively or in conjunction with surgery to reduce the size of the lesion, slow down the angiogenesis, and ultimately arrest the progression of osteolysis [11,13].

Surgical treatment is an option for patients who develop or are likely to develop pathological fractures, dysfunction, and severe pain. Resection of the affected bone and joint reconstructions with modular prosthesis systems are more prominent [13]. The patient was informed about the treatment options. Finally, pharmaceutical treatment methods were chosen as the initial treatment. The patient was treated with non-steroidal anti-inflammatory drugs, supplementation of vitamin D, and bisphosphonate. During the third-month follow-up, the patient’s symptoms were relieved. Plain radiographs showed no progression of the disease. Therefore, the patient’s medical status was discussed again in the multidisciplinary council, and her treatment was planned by pharmaceutical methods. The patient was clinically followed up for one and a half years, and no progress of the disease was noted. There was no improvement in all knee functions of the patient.

## Conclusions

Although GSD is a rare and idiopathic disorder, in the presence of lytic lesions on the musculoskeletal system, this disease must be on the diagnosis list after excluding possible disorders. Vitamin D and anti-inflammatory drugs should be considered if the disease is localized around the knee, and favorable results can be obtained in these patients. Further studies are needed to understand the disease course better as there are several challenges in the diagnosis and treatment process.
